# Complete genome sequence of *Caballeronia* sp. strain A2-2 isolated from rice paddy soil

**DOI:** 10.1128/mra.00654-26

**Published:** 2026-08-06

**Authors:** Gi Uk Hong, Ye Jin Gwak, Si Chul Kim, Hyo Jung Lee

**Affiliations:** 1 Department of Biological Science, Kunsan National University 65450, Gunsan, Republic of Korea; DOE Joint Genome Institute, Berkeley, California, USA

**Keywords:** *Caballeronia*, strain A2-2, complete genome, rice paddy soil

## Abstract

We report the genome sequence of *Caballeronia* sp. strain A2-2, isolated from rice paddy soil in the Republic of Korea. PacBio HiFi sequencing produced three chromosomes and three plasmids totaling 7,805,734 bp with 60.71% GC content and 7,259 predicted genes, including candidates for plant-associated stress and nutrient metabolism.

## ANNOUNCEMENT

Plant growth-promoting bacteria contribute to nutrient acquisition, phytohormone signaling, and stress tolerance in host plants (1). Members of the genus *Caballeronia* occur in environmental and plant-associated habitats and have been reported from soil and rhizosphere-related systems as plant growth-promoting (1, 2). Here, we report the genome sequence of *Caballeronia* sp. strain A2-2, which was isolated from rice paddy soil.

*Caballeronia* sp. strain A2-2 (NNIBR202511BA3100) was isolated from rice paddy soil collected on 12 June 2025, in Gyeongsangnam-do, Republic of Korea (35.5458°N, 128.4006°E). The soil suspension was serially diluted and plated on R2A agar (BD), and a colony was repeatedly streaked to pure culture. The single colony was inoculated and cultivated on R2A medium (BD) at 30°C for 3 days. Genomic DNA was extracted using the Wizard Genomic DNA Purification Kit (Promega) according to the manufacturer’s instructions. Genomic DNA was sheared with a Megaruptor 3 (Diagenode), size-selected with AMPure PB magnetic beads, and prepared with the SMRTbell Prep Kit 3.0 (Pacific Biosciences) according to the manufacturer’s protocol.

Whole-genome sequencing was performed on a PacBio Sequel II system. PacBio HiFi sequencing generated 264,953 reads totaling 1,597,888,681 bp, with an average read length of 6,031 bp, a read N50 of 6,614 bp, and a read N90 of 3,727 bp. *De novo* assembly was performed with Flye v2.9.4 using the --pacbio-hifi option, and circularity was determined from the Flye repeat-graph topology (3). Flye removed redundant terminal overlaps, and no independent overlap validation or sequence rotation was performed. The assembly comprised six circular contigs totaling 7,805,734 bp, with a GC content of 60.71%. The contigs were designated as three putative chromosomes of 3,455,682, 1,590,018, and 1,587,408 bp and three putative plasmids of 698,130, 315,739, and 158,757 bp. Read mapping with pbmm2 v1.14.0 yielded a mean depth of 204.4×, and BUSCO v5.5.0 (bacteria_odb10) indicated 100% completeness (4).

Genome annotation was performed with the National Center for Biotechnology Information (NCBI) Prokaryotic Genome Annotation Pipeline (PGAP) v6.10 using the best-placed reference protein set and GeneMarkS-2+ (5). PGAP predicted 7,259 total genes, including 7,090 protein-coding genes, 7,178 total coding sequences (CDSs), 81 RNA genes, 62 transfer RNAs (tRNAs), 5 complete ribosomal RNA (rRNA) gene operons (5S, 16S, and 23S), 4 non-coding RNAs, 88 pseudogenes, and 6 CRISPR arrays. The contig-level assembly and annotation features are summarized in Table 1.

**TABLE 1:** Genome features of *Caballeronia* sp. strain A2-2

Genome feature	Chromosome 1	Chromosome 2	Chromosome 3	Plasmid 1	Plasmid 2	Plasmid 3
Size (bp)	3,455,682	1,590,018	1,587,408	698,130	315,739	158,757
GC content (%)	61.31	59.73	60.63	60.48	61.47	58.01
Total CDSs	3,188	1,459	1,457	642	283	149
Protein-coding CDSs	3,168	1,423	1,442	636	278	143
Pseudogenes	20	36	15	6	5	6
rRNA genes	12	0	3	0	0	0
tRNA genes	56	1	4	1	0	0
CRISPR arrays	2	1	2	1	0	0

Taxonomic analyses using the EzBioCloud server (6), the EzBioCloud average nucleotide identity (ANI) Calculator (7), and the Genome-to-Genome Distance Calculator (8) showed that strain A2-2 shared 98.42% 16S rRNA gene sequence similarity, 82.25% ANI, and 25.2% digital DNA-DNA hybridization with its closest type strain, *Caballeronia novacaledonica* LMG 28615^T^ (GCF_900258035.1). These values were below species-level thresholds, suggesting that A2-2 may represent a novel species within the genus *Caballeronia* (9).

Manual inspection of PGAP product annotations, without additional annotation software, identified predicted homologs of ACC deaminase, urease- and urea transport-associated proteins, a NifQ-family protein, tryptophan biosynthesis enzymes, siderophore-associated proteins, a PhzF-family protein, and trehalose/glycogen metabolism enzymes. These candidates suggest possible plant-associated nutrient and stress-response traits, but their functions and plant growth-promoting effects require experimental validation (1).

## Data Availability

This Whole Genome Shotgun project has been deposited at DDBJ/ENA/GenBank under accession number JBVFIR000000000. The version described in this paper is JBVFIR010000000. The raw sequencing reads have been deposited in the NCBI Sequence Read Archive under accession number SRR38740093. The BioProject and BioSample accession numbers are PRJNA1420953 and SAMN55284225, respectively. The strain A2-2 has been deposited at Nakdonggang National Institute of Biological Resources (NNIBR) as NNIBR202511BA3100.
